# Supplementary material for: Introducing gold-standard essential gene datasets for Pseudomonas aeruginosa to enhance Tn-Seq analyses
Source: PLoS Comput Biol. 2026 Feb 9;22(2):e1013945. doi: 10.1371/journal.pcbi.1013945 (PMC12912699; doi:10.1371/journal.pcbi.1013945)
Supplement: S1 Fig — (A) Number of EGs identified with the HMM method and seven types of normalization, considering either only essential category or essential plus Growth Defect categories [Es + GD]. The proportions of low-confidence and ambiguous categories are shown. (B) Fold-enrichment of gold-standard genes recovered by each method (bars), with statistical significance indicated by p-value codes (*** p < 1 × 10 ⁻ ¹⁰, **p < 1 × 10 ⁻ ⁵, *p < 1 × 10 ⁻ 3, ns = not significant). Recall values are shown as diamond markers on the secondary axis. Colors correspond to the gold-standard datasets and the strain (WT or delta); in red the set containing the 84 core EGs for PA14 WT, in blue the set containing the 115 gold-standard genes for PA14 WT and in green the set containing the 84 core EGs for PA14 ΔoprD. (DOCX) [file pcbi.1013945.s001.docx]

# **Supplementary information**

**
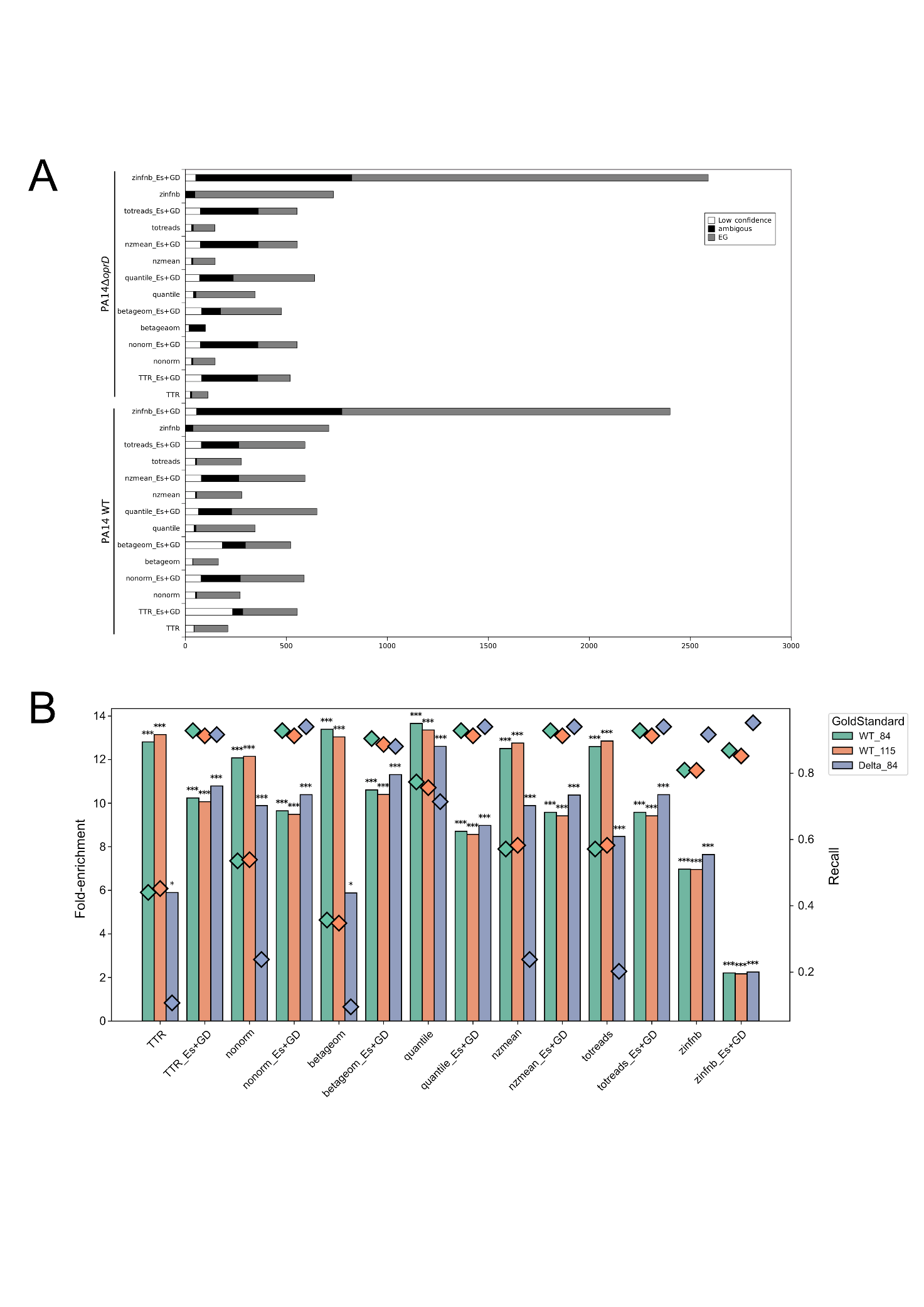
**

**S1 Fig: Impact of normalization in determining essential genes. (A)** Number of EGs identified with the HMM method and seven types of normalization, considering either only essential category or essential plus Growth Defect categories [Es+GD]. The proportions of low-confidence and ambiguous categories are shown. **(B)** Fold-enrichment of gold-standard genes recovered by each method (bars), with statistical significance indicated by p-value codes (*** p < 1×10⁻¹⁰, **p < 1×10⁻⁵, *p < 1×10⁻3, ns = not significant). Recall values are shown as diamond markers on the secondary axis. Colors correspond to the gold-standard datasets and the strain (WT or delta); in red the set containing the 84 core EGs for PA14 WT, in blue the set containing the 115 gold-standard genes for PA14 WT and in green the set containing the 84 core EGs for PA14 Δ*oprD*.
