# Supplementary material for: Introducing gold-standard essential gene datasets for Pseudomonas aeruginosa to enhance Tn-Seq analyses
Source: PLoS Comput Biol. 2026 Feb 9;22(2):e1013945. doi: 10.1371/journal.pcbi.1013945 (PMC12912699; doi:10.1371/journal.pcbi.1013945)
Supplement: S1 Table — (DOCX) [file pcbi.1013945.s002.docx]

# **Supplementary information S1**

**Table 1: Impact of parameters on providing sets of essential genes.**

|  | **^a^EG** | | **GOLD_84** | | **GOLD_115** |
| --- | --- | --- | --- | --- | --- |
|  | PA14WT | PA14∆*oprD* | PA14WT | PA14∆*oprD* | PA14WT |
| **GUMBEL_sum** | 273 | 184 | 44 | 26 | 62 |
| GUMBEL_mean | 271 | 182 | 44 | 25 | 62 |
| HMM_sum | 162 | 100 | 31 | 8 | 41 |
| **HMM_mean** | 184 | 108 | 32 | 10 | 43 |
| HMM_GD_sum | 517 | 477 | 77 | 75 | 102 |
| **HMM_GD_mean** | 565 | 520 | 77 | 76 | 104 |
| HMM_mean_loess | 210 | 111 | 37 | 9 | 52 |
| HMM_mean_GD_loess | 554 | 519 | **78** | **77** | **105** |

^a^The “EG” column indicates the number of essential genes identified by each method for PA14WT and PA14 Δ*oprD*. The columns “GOLD_84” and “GOLD_115” show the number of EGs overlapping with the gold-standard datasets. Note that the GOLD_115 dataset, which corresponds to PA14 WT, was not applied to PA14 Δ*oprD*. Default parameters and better values are highlighted in bold.
