# Supplementary material for: Introducing gold-standard essential gene datasets for Pseudomonas aeruginosa to enhance Tn-Seq analyses
Source: PLoS Comput Biol. 2026 Feb 9;22(2):e1013945. doi: 10.1371/journal.pcbi.1013945 (PMC12912699; doi:10.1371/journal.pcbi.1013945)
Supplement: S1 Text — (DOCX) [file pcbi.1013945.s003.docx]

# **Supplementary information S1**

## 1 - Impact of parameters on identifying essential genes

In TRANSIT2, there are two ways to handle replicates by averaging the read-counts (--r Mean) and by summing read counts across datasets (--r Sum). By default, Gumbel program sums read counts and HMM uses the mean counts. HMM proposes to perform LOESS correction (-l) for removing possible genomic position bias, which is turned off by default. Table 1 shows that the numbers of EGs and gold-standard genes identified remained very similar regardless of the replicate handling method and the use of the LOESS option for both PA14WT and PA14Δ *oprD* conditions. The default replicate handling options, *Sum* for Gumbel and *Mean* for HMM, yielded slightly better results. Additionally, enabling the LOESS option in HMM provided a minor improvement.
