# Supplementary material for: Introducing gold-standard essential gene datasets for Pseudomonas aeruginosa to enhance Tn-Seq analyses
Source: PLoS Comput Biol. 2026 Feb 9;22(2):e1013945. doi: 10.1371/journal.pcbi.1013945 (PMC12912699; doi:10.1371/journal.pcbi.1013945)
Supplement: S4 Fig — Each vertical bar represents a distinct set of genes, and the plot highlights the overlapping gene sets. (DOCX) [file pcbi.1013945.s006.docx]

**S4 Fig: Upset plot displaying the intersections of the essential genes sets obtained from the seven normalizations with the HMM method of TRANSIT2 for the PA14Δ*oprD* condition.** Each vertical bar represents a distinct set of genes, and the plot highlights the overlapping gene sets.

**
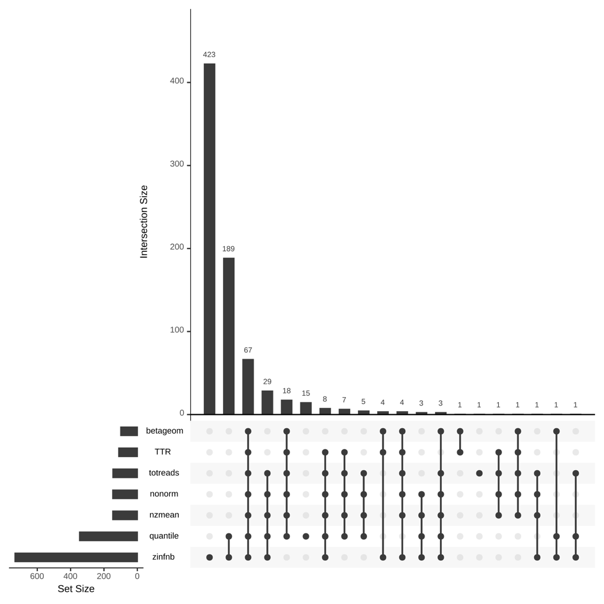
**
