# Supplementary material for: Introducing gold-standard essential gene datasets for Pseudomonas aeruginosa to enhance Tn-Seq analyses
Source: PLoS Comput Biol. 2026 Feb 9;22(2):e1013945. doi: 10.1371/journal.pcbi.1013945 (PMC12912699; doi:10.1371/journal.pcbi.1013945)
Supplement: S2 Text — (DOCX) [file pcbi.1013945.s008.docx]

# **Supplementary information S2**

## 2 - Impact of normalization on identifying essential genes

An important step in Tn-Seq analysis is the normalization of read counts as it ensures that other sources of variability are not mistakenly interpreted as real differences in datasets. TRANSIT2 offers several types of normalization.

- *Totreads:* normalizes datasets by total read-counts and scales them to have the same mean over all counts.
- ***TTR*** (Trimmed Total Reads): normalizes datasets by the total read-counts but trims top and bottom 5% of read-counts.
- *Nzmean:* normalizes datasets to have the same mean over the non-zero sites.
- *Quantile:* normalizes datasets using the quantile normalization method described by Bolstad et al. [1].
- *Betageom:* normalizes the datasets to fit an “ideal” Geometric distribution with a variable probability parameter p, particulary useful for datasets with a large skew.
- *Zinfnb:* fits a zero-inflated negative binomial model and then divides read-counts by the mean.
- ***Nonorm:*** applies no normalization

We tested the seven types of normalization proposed by TRANSIT2 (--n argument) using the bowtie mapper and the HMM method for both PA14 WT and PA14 Δ*oprD*. By default, HMM uses *TTR* normalization, which is the recommended normalization method in most cases as it accounts for differences in saturation in the context of resampling. Fig. 1 A shows that number of EGs remained relatively stable, ranging from 163 (betageom) to 346 (quantile) strictly essential genes for the PA14WT condition and from 99 (betageom) to 346 (quantile) for the PA14 Δ*oprD* condition. The exception is the zinfnb normalization which identified 710 and 733 EGs for WT and PA14 Δ*oprD*, respectively. When considering both essential and GD genes, the number of identified genes ranged from 521 (betageom) and 651 (quantile) for the WT condition, while zinfnb normalization identified 2400 genes. For PA14Δ *oprD*, the number of EGs varied from 476 (betageom) and 640 (quantile), with zinfnb normalization identifying 2,589. Fig S1 B shows that considering only genes flagged as essential leads to very variable results, from 30 (recall = 0,36) genes retrieved in GOLD_84 dataset with betageom normalization to 65 (recall = 0,77) obtained from quantile normalization for PA14 WT and from 8 (recall = 0,1) to 60 over 84 (recall = 0,71) genes for PA14Δ *oprD*. The zinfnb normalization allowing to retrieve 68 (recall = 0,81) and 77 (recall = 0,92) genes respectively for PA14WT and PA14 Δ*oprD*. However, zinfnb showed low fold-enrichment values, indicating that this normalization produced large EG lists that were not strongly enriched in gold-standard genes. When essential and GD genes were taken into account, the quantile, nonorm, nzmean and totreads normalizations allowed to identify 78 (recall = 0,93) and 79 (recall = 0,94) genes of the GOLD_84 dataset for PA14 WT and PA14 Δ*oprD*, respectively. In the PA14Δ*oprD* condition, the zinfnb normalization returned 80 genes over 84 but performed slightly poorly for the WT condition with 73 genes. The *TTR* normalization, used by default with the HMM method, returned 78 genes over 84 for the PA14 WT but only 77 genes for PA14 Δ*oprD*. Considering only strictly essential genes, between 40 (betageom) and 87 (quantile) genes of the GOLD_115 WT dataset were identified and 93 with the zinfnb normalization whereas when considering essential and GD genes, 105 (recall = 0,91) genes were retrieved with TTR, nonorm, quantile nzmean and totreads and only 98 with zinfnb. Although Zinfnb returned the higher number of EGs compared to other normalization methods, the results on gold-standard datasets were not always better and probably contains more false positives.

Intersecting the gene sets obtained from the seven normalizations showed that a large part were common, e.g. 118 EGs identified by all normalization (Fig. 2) and 443 essential and GD genes (Fig. 3) for the PA14 WT. The results were more variable for PA14Δ *oprD* with only 67 EGs in common (Fig. 4) and 452 essential and GD genes (Fig. 5). The *oprD* gene is deleted in PA14Δ*oprD* strain and so must be considered as essential in this condition as no reads will be aligned in this region. Only *quantile* and *zinfnb* lead to an essential classification for this gene whereas the five other methods classified this gene as GD.

In conclusion, the *quantile* normalization showed the best results by using only genes flagged as essential in the HMM model. However, considering essential and GD genes, the normalization method had fewer impact and led to more stable gene lists, except for *betageom* and *zinfnb* normalizations that should be avoided.

## References

1. Bolstad BM, Irizarry RA, Åstrand M, Speed TP. A comparison of normalization methods for high density oligonucleotide array data based on variance and bias. Bioinformatics. 2003;19: 185–193. doi:10.1093/bioinformatics/19.2.185

2. Rietsch A, Vallet-Gely I, Dove SL, Mekalanos JJ. ExsE, a secreted regulator of type III secretion genes in Pseudomonas aeruginosa. Proc Natl Acad Sci. 2005;102: 8006–8011. doi:10.1073/pnas.0503005102
