# Supplementary material for: Introducing gold-standard essential gene datasets for Pseudomonas aeruginosa to enhance Tn-Seq analyses
Source: PLoS Comput Biol. 2026 Feb 9;22(2):e1013945. doi: 10.1371/journal.pcbi.1013945 (PMC12912699; doi:10.1371/journal.pcbi.1013945)
Supplement: S7 Fig — Each vertical bar represents a distinct set of genes, and the plot highlights the overlapping gene sets. (DOCX) [file pcbi.1013945.s010.docx]

**S7 Fig: Upset plot displaying the intersections of the essential genes sets obtained from different statistical methods (Gumbel, HMM_GD and FiTnEss_FDR) for the PA14Δ*oprD* condition.** Each vertical bar represents a distinct set of genes, and the plot highlights the overlapping gene sets.

## 3 - Impact of statical method on providing sets of essential genes

**
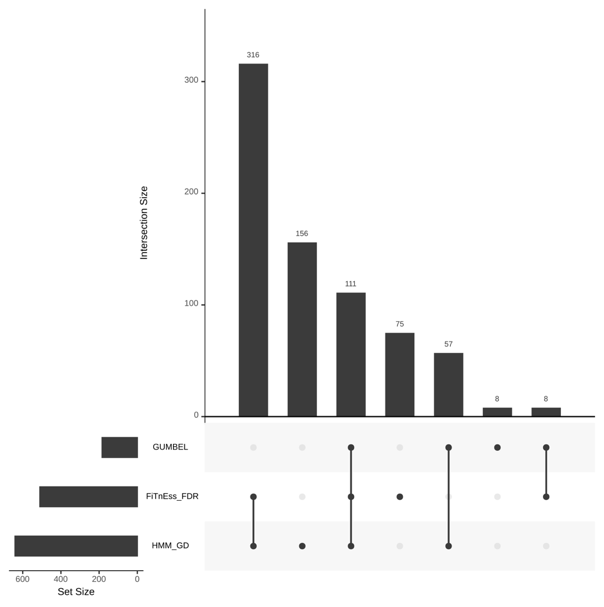
**
