# Supplementary material for: Introducing gold-standard essential gene datasets for Pseudomonas aeruginosa to enhance Tn-Seq analyses
Source: PLoS Comput Biol. 2026 Feb 9;22(2):e1013945. doi: 10.1371/journal.pcbi.1013945 (PMC12912699; doi:10.1371/journal.pcbi.1013945)
Supplement: S3 Table — (DOCX) [file pcbi.1013945.s012.docx]

## Deletion mutant construction

**S3 Table: Plasmids and primers used.**

|  | **Characteristics** | **Reference or sequence** |
| --- | --- | --- |
| **Plasmids for gene deletion** |  |  |
| **pEXG2** | Allelic exchange vector with pBR origin, gentamicin resistance, *sacB* | Arne Rietsch [2] |
| **pEXG2::*shaF*** | pEXG2 containing the Up and Down sequences (500pb) of *shaF* gene of PA14 for deletion | This work |
| **Primer** |  |  |
| ***shaF*_up_fwd** | Deletion of *shaF* gene in PA14 by Gibson Assembly, overlapping pEXG2 | cgactctagaggatccccggCAACAGACTCAATGGGATC |
| ***shaF*_up_rev** | Deletion of *shaF* gene in PA14 by Gibson Assembly, overlapping *shaF*_down | aggagtagccCTGATGGAAGCCCTGTTC |
| ***shaF*_down_fwd** | Deletion of *shaF* gene in PA14 by Gibson Assembly, overlapping *shaF*_up | cttccatcagGGCTACTCCTCTTCGGTG |
| ***shaF*_down_rev** | Deletion of *shaF* gene in PA14 by Gibson Assembly, overlapping pEXG2 | taaggtaccgaattcgagctCTACCGAAGAAGGCCTGAC |
| ***shaF*_in_fwd** | Primer to check deletion of *shaF* gene in PA14 | GAAGAACATCTCCTTGCTGA |
| ***shaF*_in_rev** | Primer to check deletion of *shaF* gene in PA14 | CTGGTACTGATCCTGATCCT |

## References

1. Bolstad BM, Irizarry RA, Åstrand M, Speed TP. A comparison of normalization methods for high density oligonucleotide array data based on variance and bias. Bioinformatics. 2003;19: 185–193. doi:10.1093/bioinformatics/19.2.185

2. Rietsch A, Vallet-Gely I, Dove SL, Mekalanos JJ. ExsE, a secreted regulator of type III secretion genes in Pseudomonas aeruginosa. Proc Natl Acad Sci. 2005;102: 8006–8011. doi:10.1073/pnas.0503005102
