# Supplementary material for: Introducing gold-standard essential gene datasets for Pseudomonas aeruginosa to enhance Tn-Seq analyses
Source: PLoS Comput Biol. 2026 Feb 9;22(2):e1013945. doi: 10.1371/journal.pcbi.1013945 (PMC12912699; doi:10.1371/journal.pcbi.1013945)
Supplement: S4 Table — (DOCX) [file pcbi.1013945.s013.docx]

**S4 Table: List of abbreviation.**

| **Abbreviation** | **Meaning** |
| --- | --- |
| EGs | Essential genes |
| FDR | False Discovery Rate |
| FiTnEss | Finding Tn-Seq Essential genes |
| FWER | Family-Wise Error Rate |
| GA | Growth Advantage (HMM category) |
| GD | Growth Defect (HMM category) |
| GOLD_115 | Gold-standard list of 115 essential genes for PA14 WT in LB |
| GOLD_84 | Gold-standard list of 84 essential genes *any P. aeruginosa strain in any growth condition* |
| HMM | Hidden Markov Model considering only EG category |
| HMM_GD | Hidden Markov Model considering only EG and Growth Defect categories |
| LOESS | Locally Estimated Scatterplot Smoothing |
| TPP | TRANSIT Pre-Processor |
| TTN-Fitness | Method correcting Himar1 insertion bias |
| ZINB | Zero-Inflated Negative Binomial |
