# Supplementary material for: Introducing gold-standard essential gene datasets for Pseudomonas aeruginosa to enhance Tn-Seq analyses
Source: PLoS Comput Biol. 2026 Feb 9;22(2):e1013945. doi: 10.1371/journal.pcbi.1013945 (PMC12912699; doi:10.1371/journal.pcbi.1013945)
Supplement: Attachment — Submitted filename: PCOMPBIOL-D-25-01258_Reply to reviewers.docx [file pcbi.1013945.s015.docx]

**Dear Editor,**

**We appreciate that you have considered our work to be suitable for peer review and for potential publication in PLOS Computational Biology, provided that all issues raised by the reviewers are adequately addressed. As requested, we have paid close attention to the comments of Reviewers #2 and #3.**

**We would like to thank the referees for the interest they have shown in our work, which aims to enable researchers to assess the quality of their Tn-Seq results and thereby promote consistency and harmonization across studies.**

**Below, we provide a point-by-point response to the reviewers’ comments. We hope that our replies, the additional supplementary experiments, and the revised manuscript will be found suitable for publication in your journal.**

**Reviewer #1:**

Defining the essential genes during TnSeq experiments is a mandatory step. Once these genes defined, the selective pressure chosen by the researchers to be applied on the saturated bank of mutants will define the genes important or essential for survival in the different conditions chosen by the scientists. As of today, no state of the art methodology has been published for finding and defining these essential genes.

**Major Comments #1**

Can groups mapping and analyzing their TnSeq data with CLC genomics use the authors approach and if yes, how?

**CLC Genomics can indeed be used for initial steps, such as mapping reads to the reference genome, obtaining insertion counts, and potentially computing RPKM (Reads Per Kilobase per Million), which is sometimes used in TnSeq analyses. However, to our knowledge, CLC Genomics does not provide the statistical functionality implemented in dedicated TnSeq tools for identifying essential genes, such as TRANSIT, for example. In addition, we chose to include only free, open-source tools in our evaluation, which can be accessed and used by the entire scientific community, unlike CLC Genomics.**

**Major Comments #2**

A final schematic to summarize their approach.

**As requested by the reviewer, we have added a figure summarizing the overview of the methodological workflow for the comparative evaluation of Tn-Seq analysis methods in *P. aeruginosa*.**

**Major Comments #3**

In the discussion, an additional emphasize on how this extensive work will be used by other researchers could be useful.

**As suggested, we have added an additional part to emphasize how our study will be useful for other researchers.**

**The gold-standard datasets and the extensive evaluation of analysis methods presented in this study are intended to serve as a resource for researchers working with Tn-Seq data. The GOLD_84 and GOLD_115 datasets provide a standardized reference for *Pseudomonas aeruginosa* enabling the assessment of new Tn-Seq data quality and the benchmarking of bioinformatic pipelines. This work can be extended to other bacterial species. In addition, our systematic comparison of mappers and parameter settings demonstrates how methodological choices affect results, thereby helping researchers select the most appropriate tools and optimize their analyses. Together, these resources facilitate reproducibility, consistency, and informed decision-making in Tn-Seq studies, and they can serve as a starting point for developing and improving Tn-Seq analysis tools.**

**Minor comments #1**

More explanation for the use of the Delta-oprD strain could be helpful for the reader.

**As mentioned in our manuscript, the starting point of our study was to establish a list of essential genes specifically for the PA14 Δ*oprD* strain, in comparison with the PA14 WT background, in order to better understand the mechanisms underlying the increased virulence associated with the loss of OprD. To address this question, we first generated a PA14 Δ*oprD* Tn insertion bank that required validation prior to any in-depth analyses, and the work presented here constitutes this initial validation step.**

**Minor comments #2**

Line 169: remove the (…), be more specific or add a reference

**References have been added to the manuscript. Details about the pre-processing steps (adapter trimming, read filtering, etc.) have been provided in the Materials and Methods section under the subsection “Analysis pre-processing steps”. We have added this clarification in the revised version of the manuscript as follows: “After the pre-processing steps, including adapter trimming and read filtering (see the Materials and Methods section for further details).**

**Reviewer #2:**

The authors performed a systematic study of commonly used analysis tools for Tn-Seq, TRANSIT2 and FiTnEss, from which they offer suggestions for parameter settings. They also provide two lists of essential genes in P. aeruginosa that they termed to be “gold-standard” sets – one specific for the strain PA14 for grown in LB and the other applicable to any Tn-seq experiment in P. aeruginosa. The authors further generated their own Tn-Seq datasets from wild-type PA14 and an OprD-deficient strain, which they used to test the parameter settings of the various tools. While the suggestions for parameters to use for analysis would be helpful to other researchers, there are crucial issues that need to be addressed. First, the gold-standard set that they delineate is not much different than what has been previously published in the literature (Poulsen et al.) and the choices made in creating those gene sets were not well-defined. It is not clear that they are appropriate gene sets for comparing analytical tools. Moreover, the authors do not provide a comprehensive metric with which to evaluate essential gene lists, as they only consider how many of the gold-standard genes are represented, but do not have any assessment of potential false positives or false negatives. Last, there are other potential biases that need to be addressed more thoroughly and are detailed in the comments below.

**Major comments #1.**

The “gold-standard” set of essential genes is very similar to what was already published in Poulsen et al. (Dataset S6), in intersecting gene lists from the same previous studies as utilized in this manuscript (Table 2). There is not enough technical justification as to why the gold standard sets defined in the manuscript should be termed “gold standard.” The GOLD_84 set particularly seems arbitrary, in that many sets are already intersections of one another, not all possible intersections are considered (e.g. Poulsen CORE could be defined using FDR and/or FWER thresholds), and some sets have more confidence than others (e.g. Poulsen FWER and FDR). Thus, the term “gold standard” is not well-defined and is overselling the novelty of the gene sets. The manuscript needs justification for the suitability of the gene sets for comparing analysis methods by providing explanations for why the authors chose to intersect the gene sets that they used and why they chose to take an intersection rather than a union of gene sets.

**The concept of core EGs has been introduced previously, for instance by Poulsen *et al.* [9], who analyzed nine *P. aeruginosa* strains grown in five different media. Similarly, the comparison of EGs across studies by intersecting individual EG sets has already been performed in several works, including Poulsen’s dataset S6. However, our objective was different: rather than comparing multiple studies or defining core EGs, we aimed to identify genes that are consistently classified as essential, regardless of the biological systems or statistical methods used. Such genes, being invariably detected as essential, could serve as true positives for protocol benchmarking and analytical validation.**

**We have detailed the datasets used from each previous study in this revised version of the manuscript (Table 2). We relied on the same studies as those included in Poulsen’s Dataset 6, although not exactly the same datasets. For example, we incorporated four datasets from Turner et al., whereas Dataset 6 included only Turner_PA14_init and Turner_PAO1_init. We also provided a clearer explanation of the purpose of the two reference datasets, referred to as the “gold standards and we have expanded the description of the technical choices made.**

**The following paragraph has been added:**

**“Our objective was not to generate an exhaustive catalog of EGs, but rather a reduced and reliable set that could serve as a reference for validation — a gold standard list of genes that should be consistently identified as essential across all Tn-Seq experiments. To construct this gold standard dataset, we postulated that genes identified as essential in multiple independent studies, each employing distinct biological systems, mapping strategies, and statistical methods, can be considered high-confidence EGs. Technically, intersecting EG lists from several studies allowed us to extract genes that were consistently identified as essential across different experimental and analytical frameworks.”**

**Although some datasets are considered more reliable than others (e.g., Poulsen_FWER), we assumed that EGs identified by multiple, independent biological and analytical approaches are more robust in our study context and should therefore be regarded as gold standard genes — that is, genes expected to be consistently identified as essential in any new Tn-eSeq experiment or analysis.**

**Major comments #2.**

The only metric the authors provide to evaluate the Tn-Seq tools is the percentage of gold-standard genes that are in the essential gene list. However, that metric does not consider how many genes are present in the essential gene list, which may be false positives. For example, if a tool outputs all the genes in the genome as its list of essential genes, it would capture 100% of the gold-standard genes, and would be “better” than the tools listed in this study. There is no penalty for the number of false positives given by the tool. The authors need to provide a more robust metric (or metrics) to quantify the performance of the tools with the given parameters, accounting for potential false positives, and possibly even false negatives.

**One difficulty we encountered in our study is that false positives, strictly speaking, cannot be determined, as they correspond to genes predicted as essential by a method but not biologically essential. Assessing this would require experimentally validating every predicted essential gene, which is not feasible. The same limitation applies to false negatives, which would require biologically testing all genes not identified as essential. Because of this, classical evaluation metrics such as accuracy or precision cannot be applied. However, we fully understand and agree with your comment regarding the percentage of gold-standard genes, which is a metric that does not account for the size of the predicted essential gene list.**

**To address this limitation, we introduced three complementary metrics: (1) fold-enrichment, (2) recall, and (3) a hypergeometric p-value.**

1) **Fold-enrichment, which measures the extent to which the set of predicted essential genes is enriched for gold-standard genes, was calculated as (TP/pred_size)/(gold_size/N), where TP is the number of true positives (predicted essential genes present in the gold-standard dataset), pred_size is the total number of predicted essential genes for a given method, gold_size is the total number of essential genes in the gold-standard, and N is the total number of testable genes in the genome.**
2) **Recall, defined as TP/gold_size, measures the proportion of gold-standard genes recovered by each method; note that this metric corresponds to the previously used percentage of gold-standard genes recovered.**
3) **Finally, statistical significance of enrichment was assessed using a hypergeometric test, providing a p-value indicating the likelihood of observing at least TP genes in the predicted set by chance.**

**These metrics were introduced in the revised version of the manuscript in the section “Impact of the mapper on identifying essential genes”. We also added a new subsection in the Materials and Methods entitled “Metrics and data visualization” describing the three metrics. Moreover, we updated the visualization: the radar plot in Figure 3B was replaced by a bar plot showing fold-enrichment, p-values, and recall for the different conditions. Figure S1B in the supplementary material was also revised to incorporate the fold-enrichment, p-value, and recall plot.**

**Major comments #3.**

The authors mention multiple other tools that are used in Tn-Seq analysis like Magenta, TSAS, etc but restrict themselves to only testing TRANSIT2 and FiTnEss. For a more comprehensive comparison of different methods, it would be ideal to test all available methods with different parameter settings. This could also give more insight into the reliability of the ‘gold-standard’ sets as a metric for determining the performance of a tool.

**Indeed, in the manuscript, we mentioned several tools for Tn-Seq analysis but focused on TRANSIT2 and FiTnEss. As stated, our choice was guided by several criteria: the tool should (i) be free and open-source, (ii) be regularly updated, (iii) offer multiple statistical methods and adjustable parameters, and (iv) be widely used. TnseqDiff is designed to identify conditionally essential genes, rather than directly essential genes, which was a central focus of our study. ESSENTIALS is a web-based tool that is no longer available and has not been updated for 13 years, similar to TnSeq Explorer (no update for 10 years) and Magenta (no update for 8 years). ARTIST is MATLAB-based and neither free nor open-source, which led us to exclude it from our analysis. Consequently, the set of relevant tools was reduced to TSAS, TRANSIT2, and FiTnEss.**

**We chose to include FiTnEss because it was specifically developed for *P. aeruginosa* Tn-Seq data. TRANSIT, in contrast, is very regularly updated, with TRANSIT2 representing a complete reimplementation in Python. It is also the most widely used tool for Tn-Seq data analysis, offering a graphical user interface (GUI) for users unfamiliar with command-line tools and implementing a wide range of statistical methods and adjustable parameters. TSAS was updated two years ago (version 2), but it is less widely used than TRANSIT (54 citations vs. 276) and offers fewer statistical methods and parameter options.**

**For these reasons, we focused on TRANSIT2 and FiTnEss in our study. Our aim was not to be exhaustive, but rather to propose a formal framework that can be used to evaluate other tools, parameters settings, or bacterial strains. For researchers interested in TSAS, our evaluation protocol could also be applied to this tool.**

**We provided a clearer justification for this choice in the revised version of the manuscript.**

**Major comments #4.**

The section “Impact of the mapper on identifying essential genes” has potential biases that need to be addressed. Specifically, the mapper used in the previous studies were not stated, and it is possible that bowtie yielded more of the gold standard genes than BWA simply because all the studies from which the gold standard genes were derived also used bowtie rather than BWA. The result may not be due to bias in the data rather than to actual differences in the mappers. Could the authors add which aligners were used in each of the studies in Table 2, and adjust their interpretation accordingly?

**We summarized the mappers and statistical approaches used in each previous study in Table 2. Various statistical approaches and mappers were employed (BLAST, CLC Mapper, Bowtie, Bowtie2, and BWA), ensuring that the observed results are not attributable to biases in the gold-standard datasets.**

**Major comments #5**

Also in the section “Impact of the mapper on identifying essential genes,” the authors should more comprehensively assess whether normalization techniques could adjust for differences in performance between BWA and bowtie. In Table 3, BWA seems superior to bowtie in most metrics except for skewness, and yet the authors suggest to use bowtie. Would it be possible that using alternate normalization techniques such as the “betageom” option in TRANSIT2 could account for the skewness and improve the performance using BWA? It seems that the aligner was chosen prior to adjusting any of the other parameters in the TRANSIT2 tool, and that the testing was not comprehensive in this regard.

**In the revised version of the manuscript, we provided a more detailed description of the metrics in Table 3, which allowed us to recommend the use of Bowtie rather than BWA. We also computed and added quality metrics for both Bowtie and BWA after applying the betageom and TTR normalization options available in TRANSIT2, which are presented as a new supplementary table (Table S2). The results show that the betageom option improves quality metrics for BWA. We then examined the impact of the nonorm, betageom, and TTR normalization options on essential gene identification (Table S2). These analyses further suggest that BWA generates a higher level of non-specific mappings, which may lead to an underestimation of essential genes. All of these results have been integrated and discussed in the revised version of the manuscript. Finally, we revised the conclusion of this section to better reflect the results obtained with BWA.**

**Major comments #6**

While the authors provide empirical evidence for some parameters yielding better results than others, they do not provide explanations to explain why those results occur. Could authors provide some technical conjectures as to why the parameters they chose yielded the best results?

**In the revised version of the manuscript, we provided several technical hypotheses to explain the results obtained when modifying certain parameters. For example, we substantially rewrote the section discussing the impact of the mappers and added hypotheses to explain the differences in metrics obtained with BWA and Bowtie. We also proposed explanations for why the Gumbel method consistently returned fewer essential genes than other methods in the subsection “Impact of statistical method on providing sets of essential genes.”**

**Minor comment #1**

In Figure 3b, is the delta-oprD for the 115 gene set missing?

**The Δ*oprD* dataset for GOLD_115 is not missing from Figure 3b. The GOLD_115 set serves as the gold standard specifically for PA14 WT grown in LB, whereas GOLD_84 is a more general essential-gene list applicable to any *P. aeruginosa* strain in any growth condition. Since Δ*oprD* is a mutant strain, only the GOLD_84 dataset is appropriate for use.**

**Minor comment #2**

In the section comparing bwa and bowtie, the lower bound for the density metric to assess performance of the aligner is given as 35%, while the citation used for the same gives it as 30%.

**There was a mistake in the manuscript: the correct value is 30%, not 35%. This error has been corrected in the revised version of the manuscript. Thank you for pointing it out.**

**Minor comment #3**

The language in the “Methods to determine conditional gene essentiality” section needs to be clarified. Particularly when intersections are mentioned, it would be helpful to specifically state what the intersection is between. Also, it would help to state explicitly that the genes that were not in the intersection between the essential genes in wild-type versus delta-oprD were considered to be the conditionally essential genes. Currently it seems to read that the genes in the intersection were the conditionally essential genes.

**We clarified this point in the revised version of the manuscript : « Therefore, for FiTnEss and HMM, the EG lists obtained for PA14 WT and PA14 Δ*oprD* were intersected to identify the essential genes specific to PA14 WT and PA14 ΔoprD grown in LB. Genes that were not included in the intersection of EGs between PA14 WT and PA14 Δ*oprD* were considered conditionally essential. »**

**Minor comment #4**

At the end of the section “Methods to determine conditional gene essentiality,” it states “we propose FiTnEss_FDR complemented with information retrieved from ZINB, would be an accurate and reliable method…” Could the authors explain how they came to this conclusion and practically how a researcher would “complement the information” with the other method?

**We rewrote this paragraph to better clarify our conclusions in the revised version of the manuscript :**

**Altogether, these findings showed that each method used to determine conditional gene essentiality returned its own list of EGs. Among the statistical methods, the resampling approach produced a large number of conditionally essential genes compared to the other methods and should therefore be avoided. The ANOVA and utest methods yielded very few genes, and moreover, utest did not report the *oprD* gene as conditionally essential in PA14 Δ*oprD*. Consequently, these two methods should also be avoided. Finally, the ZINB method appears to yield consistent results and can be used to determine conditional gene essentiality. Regarding the intersection-based methods, as shown previously, FiTnEss_FDR provided more robust EG identification. Here, we showed that no GOLD_84 gene was identified as conditionally essential with FiTnEss_FDR, whereas three GOLD_84 genes were detected with HMM_GD; therefore, FiTnEss_FDR should be preferred. Accordingly, we propose that FiTnEss_FDR, complemented with information retrieved from ZINB, would be an accurate and reliable method for determining conditional essential genes in *P. aeruginosa*. Indeed, ZINB, like the other statistical methods, relies on raw read counts and identifies genes showing significantly different read counts between the two conditions; however, these genes are not necessarily classified as essential. In contrast, FiTnEss_FDR considers only essential genes that are specific to one condition. Therefore, if the user aims to identify strictly conditional essential genes, we recommend using FiTnEss_FDR, and using ZINB otherwise. ZINB outputs an informative table with several values, such as the mean read count for each condition, log2 fold-change values, or gene status indicators (e.g., low numbers of TA sites in a gene). Even if the user uses the FiTnEss_FDR gene list, all the ZINB values can still help assess whether a gene can be considered conditionally essential.**

**Minor comment #5**

It would be helpful to have a table describing all the method abbreviations used in the manuscript. It would also be helpful to be consistent in referring to these abbreviations in both the figures and the text. For example, sometimes it seems that HMM is used interchangeably with HMM_GD, which can be confusing to the reader.

**We thank the reviewer for pointing out this issue. We added a table describing all method abbreviations used in the revised version of the manuscript. We also corrected instances of confusion between HMM and HMM_GD, particularly in the subsection “Impact of statistical method on providing sets of essential genes.”**

| **Abbreviation** | **Meaning** |
| --- | --- |
| EGs | Essential genes |
| FDR | False Discovery Rate |
| FiTnEss | Finding Tn-Seq Essential genes |
| FWER | Family-Wise Error Rate |
| GA | Growth Advantage (HMM category) |
| GD | Growth Defect (HMM category) |
| GOLD_115 | Gold-standard list of 115 essential genes for PA14 WT in LB |
| GOLD_84 | Gold-standard list of 84 essential genes *any P. aeruginosa strain in any growth condition* |
| HMM | Hidden Markov Model considering only EG category |
| HMM_GD | Hidden Markov Model considering only EG and Growth Defect categories |
| LOESS | Locally Estimated Scatterplot Smoothing |
| TPP | TRANSIT Pre-Processor |
| TTN-Fitness | Method correcting Himar1 insertion bias |
| ZINB | Zero-Inflated Negative Binomial |

**Reviewer #3:**

This study by Maele et al. addresses a critical need in the Pseudomonas aeruginosa research community by establishing gold-standard essential gene (EG) datasets to benchmark bioinformatics pipelines for transposon sequencing (Tn-Seq) analyses. Drawing on both literature and newly generated Tn-seq data from PA14 wild-type and ΔoprD strains grown in LB, the authors compared EG lists produced by several statistical methods implemented in TRANSIT2 and FiTnEss. They constructed a reference set of 84 EGs for P. aeruginosa, as well as a PA14-LB-specific list of 115 EGs, and assessed how effectively these genes could be identified using their datasets. Retrieval rates varied across methods, with the Hidden-Markov Model in TRANSIT2 recovering approximately 90% of gold-standard EGs and FiTnEss achieving up to 100%. These curated datasets will prove valuable for the Tn-seq community, providing objective benchmarks for evaluating analysis pipelines, thereby improving reproducibility, standardization, and essential gene identification. The study is well organized, with clear writing and effective figures. I have several suggestions to enhance clarity and expand the scope of the work.

**Major comment #1:**

The motivation for utilizing the ΔoprD mutant in Tn-seq experiments is not clear. Further, the subsequent comparison of statistical tests for identifying conditional essential genes between the wild type and ΔoprD backgrounds is somewhat confusing. Could the authors include a known essential gene that displays conditional essentiality in the ΔoprD background (but not in wild type) as a positive control to validate the statistical tests? Alternatively, have the authors performed experimental validation—for instance, by comparing gene deletions in both backgrounds, such as with shaF? Additionally, what would the authors predict if a similar comparison was conducted in another gene deletion background?

**As noted in the manuscript, the mechanism underlying the increased virulence associated with the loss of OprD in PA14 remains unclear. To begin addressing this question, our long-term objective is to identify the essential genes in the PA14 Δ*oprD* strain and to compare them with those of the PA14 WT background, as this may ultimately help reveal pathways contributing to the enhanced pathogenicity of the Δ*oprD* mutant. Before undertaking any such biological interpretation (which is beyond the scope of the present article), it was necessary to validate the quality and saturation of the Δ*oprD* transposon insertion library. The comparison of statistical approaches presented in this study therefore reflects this validation step, rather than an attempt to draw conclusions about conditional essentiality specific to the Δ*oprD* background.**

**We also appreciate the reviewer’s suggestion to include a known gene displaying conditional essentiality in the Δ*oprD* background. To our knowledge, no such gene has been described in the literature; identifying such genes is precisely one of the biological questions motivating the broader project. Importantly, the clean deletion of *oprD* in our Δ*oprD* strain provides an intrinsic positive control for conditional essentiality. Because the *oprD* locus is deleted in the Δ*oprD* strain, no reads can be mapped to the *oprD* gene, and it is therefore necessarily called “essential” in the Δ*oprD* dataset but not in the WT background. Beyond this built-in control, no additional known conditional essential gene is currently available for validating the statistical tests, which further underscores the need for the validation work presented in this manuscript.**

**In addition, gene deletion was attempted for *shaF* in both PA14 WT and PA14 Δ*oprD*. While the deletion was successfully obtained in the wild-type strain, it could not be generated in the Δ*oprD* mutant. This information has now been added to the manuscript.**

**Major comment #2:**

The authors refer to two essential gene lists from prior studies: GOLD 84 (general P. aeruginosa essential genes) and GOLD 115 (PA14-specific essential genes). While it is noted that the gene ontology profiles of these lists are similar, it would be helpful if the authors could highlight and discuss the key differences between these two sets to provide readers with more context.

**GOLD_115 is specific to the PA14 strain grown in LB medium and therefore represents a narrower, condition- and strain-specific set of essential genes. In contrast, GOLD_84 is derived from multiple *P. aeruginosa* strains grown under various conditions and thus corresponds to a more general “core” set of essential genes that are largely conserved regardless of strain or growth medium. While the overall gene ontology profiles of these two lists are similar, GOLD_115 may include genes whose essentiality is conditional on the PA14 genetic background or on growth in LB, whereas GOLD_84 reflects essential genes with broader applicability.**

**These clarifications have been added to the manuscript.**

**Major comment #3**

For these two essential gene lists, it would be useful to include a summary of the mappers and statistical approaches employed in prior studies in a table. It would be important for the authors to clarify how robust the ‘gold standard’ lists are to different analysis methods.

**We summarized the mappers and statistical approaches used in each previous study in Table 2.**

**Major comment #4**

The focus on PA14 is understandable given the availability of high-quality Tn-seq datasets, but this may limit the generalizability of the findings. Could the authors consider extending their analysis to PAO1 using publicly available Tn-seq datasets or discuss the potential for broader application of their evaluation framework to non-Pseudomonas Tn-seq studies?

**It would be possible to extend our analysis to generate a PAO1-specific gold-standard dataset using publicly available data; however, this is beyond the scope of our study. More broadly, the main contribution of our work is the evaluation workflow itself, which can be adapted to different strains, species, and experimental conditions. Researchers can use our approach to construct strain- or species-specific gold standards and to identify the most appropriate analytical methods for their own Tn-seq datasets.**

**Nevertheless, the GOLD_84 dataset includes essential genes that are conserved across *P. aeruginosa* strains and growth conditions, and it can therefore be applied to PAO1 by mapping the GOLD_84 genes to their PAO1 orthologues. We added a column containing the PAO1 locus tags in the table listing the 84 gold-standard genes, so that researchers working with PAO1 can readily use this resource.**

**Minor comment #1:**

The resolution of Figure 1A and 1B is low and should be improved for clarity.

**In response to the reviewer’s comment, Figures 1A and 1B have been regenerated at higher resolution to improve clarity.**
